# A de novo TLR7 gain-of-function mutation causing severe monogenic lupus in an infant

**DOI:** 10.1172/JCI179193

**Published:** 2024-05-16

**Authors:** Jarmila Stremenova Spegarova, Praisoody Sinnappurajar, Dalila Al Julandani, Rokas Navickas, Helen Griffin, Manisha Ahuja, Angela Grainger, Katie Livingstone, Gillian I. Rice, Fraser Sutherland, Corinne Hayes, Simon Parke, Lewis Pang, Marion R. Roderick, Mary Slatter, Yanick Crow, Athimalaipet V. Ramanan, Sophie Hambleton

**Affiliations:** 1 Translational and Clinical Research Institute, Medical School, Newcastle University, Newcastle upon Tyne, United Kingdom.; 2 Department of Paediatric Rheumatology, Bristol Royal Hospital for Children, Bristol, United Kingdom.; 3 MRC Human Genetics Unit, Institute of Genetics and Cancer, University of Edinburgh, Edinburgh, United Kingdom.; 4 Division of Evolution and Genomic Sciences, School of Biological Sciences, Faculty of Biology, Medicine and Health, University of Manchester, Manchester Academic Health Science Centre, Manchester, United Kingdom.; 5 Department of Paediatrics and; 6 Exeter Genomics Laboratory, Royal Devon University Healthcare NHS Foundation Trust, Exeter, United Kingdom.; 7 Department of Paediatric Immunology, Bristol Royal Hospital for Children, Bristol, United Kingdom.; 8 Translational Health Sciences, University of Bristol, Bristol, United Kingdom.; 9 Great North Children’s Hospital, Royal Victoria Infirmary, Newcastle upon Tyne, United Kingdom.

**Keywords:** Autoimmunity, Immunology, Lupus, Monogenic diseases, Stem cell transplantation

**To the Editor:** Childhood-onset systemic lupus erythematosus (cSLE) presents with a more severe phenotype and worse outcomes than adult-onset SLE (1). Genetic factors are understood to have a significant role in cSLE and can occur secondary to a single-gene defect, termed monogenic lupus (2). Recently, germline gain-of-function mutations of *TLR7* were shown to cause cSLE, highlighting the role of *TLR7* in driving autoimmunity (3). Here, we demonstrate a previously unreported de novo gain-of-function *TLR7* variant in a 2-year-old girl with severe SLE and outline a successful approach to disease management.

A 13-month-old female infant presented with anti-NMDA receptor encephalitis. Within 10 months, she developed a large pericardial effusion and profound hemolytic anemia and was diagnosed with cSLE. Shortly afterward, she developed inflammatory vasculitis with sudden-onset status epilepticus. Each presentation was life-threatening and required substantial immunosuppression (details in Supplemental Figure 1 and Supplemental Table 1; supplemental material available online with this article; https://doi.org/10.1172/JCI179193DS1). Despite clinical improvement, both her interferon-stimulated gene (ISG) signature and neutrophil transcriptional signature remained pathologically elevated (Figure 1, A and B and Supplemental Methods).

The very early onset of recurrent, life-threatening immune dysregulation raised suspicion for an inborn error of immunity (IEI), but whole-exome sequencing (Figure 1C) was negative for known pathogenic variants. However, comparison of proband and parental sequences revealed a previously unreported de novo heterozygous missense mutation in *TLR7*, (c.800C>T, p.P267L), confirmed by Sanger sequencing (Figure 1D). The substitution of this highly conserved proline residue in the leucine-rich repeat (LLR9) ectodomain, lying only 3 residues from the recently described TLR7^Y264H^ gain-of-function variant (3), was predicted deleterious.

The encoded product, TLR7, is part of a canonical defense system, linking innate and adaptive immunity. TLR7 predominantly recognizes viral single-stranded RNA in the endosomes of hematopoietic cells. Its signaling leads to activation of interferon regulatory factors to induce interferon synthesis and of NF-κB and MAPK pathways to activate the transcription of proinflammatory cytokines. While loss-of-function TLR7 variants predispose to severe viral infection (4), TLR7 gain of function was recently identified in several young children with SLE (3), and polymorphisms affecting *TLR7* expression are also recognized to influence adult-onset SLE risk (5).

We hypothesized that TLR7 p.P267L causes cSLE through a gain-of-function mechanism. Accordingly, stimulation of patient cells by TLR7/8 ligand CL097 in vitro led to elevated transcriptional activation of proinflammatory cytokines, TNF-α, IL-1b, and IL-6 (Figure 1E and Supplemental Methods), while CD62L shedding was unaffected (Supplemental Figure 2 and Supplemental Methods). Standard PBMC immunophenotyping was normal, other than reduced B cell numbers reflecting prior B cell–depleting therapy (Supplemental Figure 3 and Supplemental Methods). However, TLR7 protein expression was increased, especially in B cells, monocytes, and dendritic cells (Figure 1, F and G, and Supplemental Methods).

To further evaluate the variant’s effect on protein function, we first performed transient transfection of WT or mutant TLR7 into HEK293T cells and documented equivalent protein expression (Figure 1H). Utilizing a cotransfected NF-κB reporter system, TLR7^P267L^ generated significantly higher dose-dependent NF-κB signaling than WT TLR7 in response to two different TLR7-specific agonists (Figure 1I and Supplemental Methods). Taken together, these results confirm our hypothesis of gain of function for the TLR7^P267L^ variant and imply that increased protein abundance in patient leukocytes (perhaps itself interferon driven) may amplify the effect of higher levels of signaling activity.

Following diagnosis of IEI, JAK inhibitor therapy was introduced to the patient to act as a bridge to a curative procedure in the form of a conditioned maternal TCRαβ/CD19-depleted haploidentical hematopoietic stem cell transplant, which was well tolerated. Nine months after transplant, SARS-CoV-2 infection triggered an autoimmune hemolytic anemia, requiring transfusion and immunomodulation. Over the following 8 months the patient remained positive for red cell antibodies by direct antiglobulin testing on sirolimus and physiological dose corticosteroid, with evidence of smoldering hemolysis and mildly raised ISG signature (final time point in Figure 1, A and B) in the context of 90% donor chimerism. Nonetheless she has remained systemically well and made excellent progress in terms of immune reconstitution, growth, and neurodevelopment (details in Supplemental Figure 1).

Despite intense research, understanding of the etiology of SLE remains incomplete (6). The recognition of rare IEI in patients with cSLE may provide valuable insights into disease mechanism and suggest targets for precision medicine. Our present findings reinforce the importance of innate immunity in SLE pathogenesis while highlighting the curative potential of hematopoietic stem cell transplant in TLR7 gain of function.

## Supplementary Material

Supplemental data

Unedited blot and gel images

Supporting data values

## Figures and Tables

**Figure 1:**
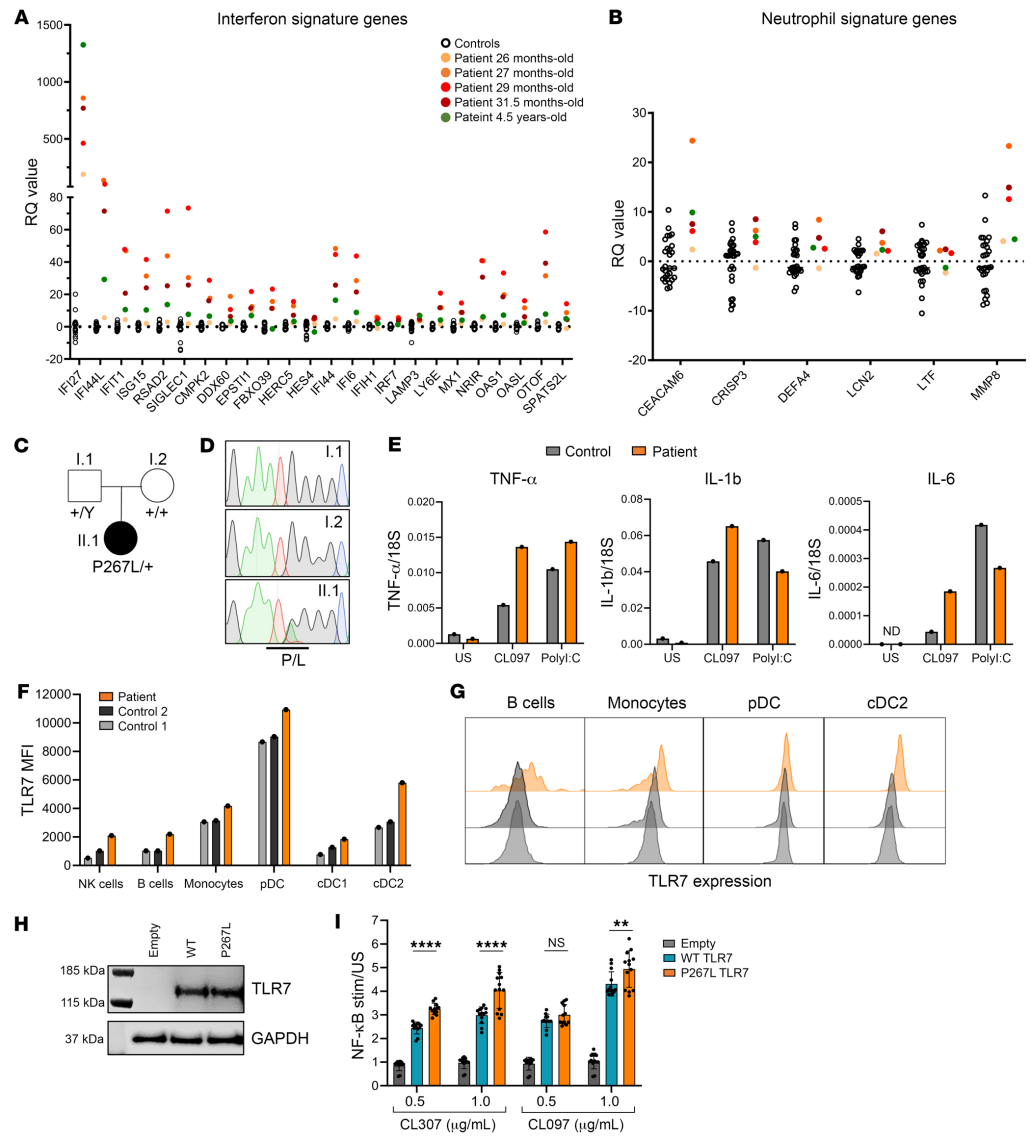
TLR7 gain-of-function mutation in a young child with severe SLE. (**A**) Persistently pathologically elevated interferon-stimulated gene and (**B**) neutrophil signature gene transcripts in patient at indicated time points. The *y* axis shows transcript abundance in arbitrary units (relative quantification [RQ] values) for indicated genes. (**C**) Pedigree. (**D**) Capillary sequencing of PCR amplicons. P, proline substituted by L, leucine. (**E**) Increased transcription of proinflammatory cytokines TNF-α, IL-1b, and IL-6 in patient PBMCs stimulated with TLR7/8 ligand CL097 or polyI:C. US, unstimulated. (**F**) Increased TLR7 protein expression in patient PBMC subsets quantitated by (**G**) flow cytometry. Representative of 2 independent experiments. (**H**) TLR7 protein expression detected by immunoblotting of transfected HEK293T cells 48 hours after transfection. (**I**) NF-κB activity by dual luciferase assay after TLR7 plasmid transfection into HEK293T cells and treatment with indicated TLR7 ligands. Luminescence signal normalized to unstimulated cells from 4 independent experiments. Two-way ANOVA, **P < 0.01, ****P < 0.0001. Data represent mean ± SD.

## Version 1 05/16/2024

In-Press Preview

## Version 2 07/01/2024

Electronic publication
