# Supplementary material for: A de novo TLR7 gain-of-function mutation causing severe monogenic lupus in an infant
Source: J Clin Invest. 2024 May 16;134(13):e179193. doi: 10.1172/JCI179193 (PMC11213501; doi:10.1172/JCI179193)
Supplement: Supplemental data [file jci-134-179193-s120.pdf]

Supplemental Material

A de novo TLR7 gain-of-function mutation causing severe monogenic lupus in an infant.

Jarmila Stremenova Spegarova[1], Praisoody Sinnappurajar[2], Dalila Al Julandani[2], Rokas Navickas[1], Helen Griffin[1], Manisha Ahuja[1], Angela Grainger[1], Katie Livingstone[3], Gillian I Rice[4], Fraser Sutherland[3], Corinne Hayes[5], Simon Parke[5], Lewis Pang[6], Marion R Roderick[7,8], Mary Slatter[9], Yanick Crow[3], Athimalaipet V Ramanan[2,8], Sophie Hambleton[1,9]

[1]Translational and Clinical Research Institute, Medical School, Newcastle University, Newcastle upon Tyne, UK. [2]Department of Paediatric Rheumatology, Bristol Royal Hospital for Children, Bristol, UK. [3]MRC Human Genetics Unit, Institute of Genetics and Cancer, University of Edinburgh, Edinburgh, UK. [4]Division of Evolution and Genomic Sciences, School of Biological Sciences, Faculty of Biology, Medicine and Health, University of Manchester, Manchester Academic Health Science Centre, Manchester, UK. [5]Department of Paediatrics and [6]Exeter Genomics Laboratory, Royal Devon University Healthcare NHS Foundation Trust, Exeter, UK. [7]Department of Paediatric Immunology, Bristol Royal Hospital for Children, Bristol, UK. [8]Translational Health Sciences, University of Bristol, Bristol, UK. [9]Great North Children's Hospital, Royal Victoria Infirmary, Newcastle upon Tyne, UK.

Correspondence to: Professor Sophie Hambleton, Newcastle University Translational & Clinical Research Institute, Framlington Place, Newcastle upon Tyne, NE2 4HH, UK, sophie.hambleton@newcastle.ac.uk, +44 191 208 7139

Funding: Work in the Hambleton laboratory was supported by Wellcome (award number 207556_Z_17_A). YJC acknowledges funding from the European Research Council (786142 E-T1IFNs), MRC UK (core grant, MC_UU_00035/11) and ANR France (ANR-10-IAHU-01 and ANR-23-CE15-0015-02)

Conflict of Interest: SH declares research funding from Pharming and has received honoraria for consultancy or teaching from Takeda, CSL Behring, Pharming, Hitachi Vantara and Videregen.

Supplemental Material Content

Supplementary Methods

Supplementary References

Supplementary Figure S1. Patient’s progress timeline.

Supplementary Table 1. Patient’s laboratory values.

Supplementary Figure S2. Normal CD62L shedding in patient.

Supplementary Figure S3. Deep immunophenotyping of patient’s PBMC.

Supplementary Methods

Ethical approvals

Written consent was obtained from the patient's parents and healthy controls as part of the ethically approved project REC reference 20/NE/0044.

Genetic Analysis

Family trio genomic DNA was subjected to whole-exome sequencing and analysed by standard methods. Revealed candidate disease-causing variant *TLR7* was confirmed by Sanger sequencing using primers pair: Forward: 5'-CAGCCGTCCCTACTGTTTTG-3', Reverse: 5'-CATGCTGAAGAGAGTTACTGTGT-3' by Source Bioscience.

TLR7 p.P267L is absent from GnomADv4, 1000Genomes and dbSNP v155 population databases. Prediction scores: SIFT = 0.02, deleterious; PolyPhen = 0.973, probably damaging; CADD score v1.7 = 23.4; vertebrate PhyloP conservation score = 7.564, high.

Quantitative RT-PCR of signature genes

Whole blood was collected in PAXgene tubes (PreAnalytiX). Total RNA was extracted from samples using the PAXgene blood RNA kit (PreAnalytiX) according to manufacturer's instructions. The analysis of 24 genes and 3 housekeeping genes was conducted using the NanoString customer designed Code-Sets according to the manufacturer's recommendations (NanoString Technologies). One hundred nanograms of total RNA was loaded for each sample. Agilent Tapestation was used to assess the quality of the RNA. Data were processed with nSolver software (NanoString Technologies). The data were normalized relative to the internal positive and negative calibrators, the three reference probes, and healthy control samples. The median of the 24 probes for each of 29 healthy control samples was calculated. The mean NanoString score of 29 healthy controls + 2 SD of the mean was calculated. Scores above this value were designated as positive. ISG score cut-off 2.758; Neutrophil score cut-off 6.088. Probes were IFI27, IFI44L, IFIT1, ISG15, RSAD2, SIGLEC1, CMPK2, DDX60, EPSTI1, FBXO39, HERC5, HES4, IFI44, IFI6, IFIH1, IRF7, LAMP3, LY6E, MX1, NRIR, OAS1, OASL, OTOF, and SPATS2L. Reference probes were HPRT1, NRDC, and OTUD5. Generation of an interferon signature by qPCR of a 6-gene panel is as described in Rice GI et al. (Rice GI, et al. Assessment of interferon-related biomarkers in Aicardi-Gouti.res syndrome associated with mutations in TREX1, RNASEH2A, RNASEH2B, RNASEH2C, SAMHD1, and ADAR: a case-control study. *Lancet Neurol.* 2013;12(12):1159–69).

Quantitative RT-PCR of pro-inflammatory cytokines

Patient's and healthy control PBMC were stimulated for four hours at 37°C with 1ug/ml TLR7/8 ligand CL097 (InvivoGen, tlrl-c97) or 25ug/ml PolyI:C (Merck, Sigma-Aldrich, P1530) at 37°C in complete RPMI-1640 culture medium (Sigma Aldrich, R0883) supplemented with 10% (v/v) foetal calf serum (FCS, Gibco, 10270-106), 1% (v/v) Penicillin/Streptomycin (100 U/mL and 100 µg/mL respectively; Sigma Aldrich, P0781) and 1% (v/v) L-Glutamine (2 mM; Sigma Aldrich, G7513), referred as RPMI10. RNA was extracted using ReliaPrepTM RNA Cell Miniprep System (Promega, Z6011), and reverse transcribed by Invitrogen SuperScript™ III Reverse Transcriptase kit (Thermo Fisher Scientific, 2520318) according to the manufacturers' instructions. cDNA templates were subjected to quantitative RT-PCR with a TaqManGene Expression Master Mix (Applied Biosystems, Thermo Fisher Scientific) according to the manufacturer's instructions. The primers and related probes for TNF-alpha, IL-6, IL-1b and 18S were designed by Roche Universal Probe Library System Assay Design.

CD62L (L-selectin) shedding assay

CD62L (L-selectin) is detected on neutrophils prior to stimulation and should be absent (shed) after stimulation. Fresh whole EDTA blood was stimulated with 2ug/ml Phorbol-12-Myristate-13 Acetate (PMA, VWR) as a positive control bypassing the TLR's, 100ug/ml Lipopolysaccharide (LPS, TLR4 ligand, SLS), or 12ug/ml TLR7/8 ligand CL097 (InvivoGen, tlrl-c97), and incubated at 37 °C for 1hour. Subsequently cells were washed and stained with isotype control or CD62L FITC antibody (DREG-56, BD Biosciences) for 15min at RT. Cells were lysed using FACS lyse buffer (Becton Dickinson) for 15min at RT, washed and acquired on a FACS Canto flow cytometer (Beckman Coulter). Data were analysed by FlowJo V10 (BD Biosciences).

Immunophenotyping and TLR7 protein expression in patient's PBMC

Peripheral blood mononuclear cells (PBMC) were isolated from EDTA blood samples using Lymphoprep (StemCell Technologies, 07851) density gradient centrifugation as per manufacturer's instructions. PBMC were thawed at 37°C, transferred into pre-warmed complete RPMI10 culture medium, pelleted by centrifugation, resuspended in RPMI10 and left to rest at 37°C for 1.5 hours.

For cell surface markers, cells were stained with a cocktail of antibodies in FACS buffer (PBS + 2% FCS) for 30min at room temperature (RT) in the dark. Cells were washed and resuspended in FACS buffer, 7AAD viability dye (Biolegend, 1:20) was added, and cells were acquired on a BD Symphony A5 flow cytometer (BD Biosciences). Used anti-human flow cytometry antibodies: TCRyd FITC (B1, BioLegend, 1:20), CD127 PerCP-Cy5.5 (A019D5, BioLegend, 1:50), CCR6 PE (11A9, BD Biosciences, 1:50), CD56 PE-CF594 (NCAM16.2, BD Biosciences, 1:100), CCR7 Pe-Cy7 (G043H7, BioLegend, 1:50), CXCR5 AF647 (J252D4,

BioLegend, 1:10), PD-1 AF700 (EH12.2H7, BioLegend, 1:50), CD45RO APC-Cy7 (UCHL1, BioLegend, 1:50), CXCR3 BV421 (1C6, BD Biosciences, 1:33), CD28 BV480 (CD28.2, BD Biosciences, 1:50), CD57 BV605 (QA17A04, BioLegend, 1:100), CD16 BV650 BD (3G8, Biosciences, 1:100), CD95 BV711 (DX2, BD Biosciences, 1:50), HLA-DR BV750 (L243, BioLegend, 1:50), CD4 BV785 (SK3, BioLegend, 1:100), CD3 BUV395 (UCHT1, BD Biosciences, 1:50), CD8 BUV496 (RPA-T8, BD Biosciences, 1:100), CD25 BUV737 (2A3, BD Biosciences, 1:100).

For intracellular markers staining, cells were pre-stained for 30min at RT in the dark with fixable viability dye Zombie Yellow (Biolegend, 1:200), and anti-human antibodies CD27 PE-Dazzle (M-T271, BioLegend, 1:20), CD20 BV510 (2H7, BioLegend, 1:100), CD16 BV650 (3G8, BD Biosciences, 1:100), IgD BUV496 (IA6-2, BD Biosciences, 1:50), CD38 BUV661 (HIT2, BD Biosciences, 1:20) and CD14 BUV737 (M5E2, BD Biosciences, 1:100). Cells were washed with FACS buffer and fixed with 2% paraformaldehyde (ThermoFisher Scientific) for 15min at RT. Subsequently, cells were washed with PBS, permeabilized with 1x BD Perm/Wash buffer (BD Biosciences) according to manufacturer's instruction and stained with antibodies for 30min at 4°C: CD3 FITC (SK7, BD Biosciences, 1:50), CD56 FITC (NCAM16.2, BD Biosciences, 1:50), CD19 PerCP-Cy5.5 (HIB19, BioLegend, 1:20), TLR7 PE (S18024F, BioLegend, 1:20), CD1c PE-Cy7 (L161, BioLegend, 1:50), CD303 APC (201A, BioLegend, 1:33), CD11c BV421 (B-ly6, BD Biosciences, 1:50), HLA-DR BV711 (L243, BioLegend, 1:50), IgG BV786 (G18-145, BD Biosciences, 1:100) and CD123 BUV395 (7G3, BD Biosciences, 1:50). Cells were washed and acquired on a BD Symphony A5 flow cytometer (Beckman Coulter). Data were analysed by FlowJo V10 (BD Biosciences).

Site directed mutagenesis

To create a plasmid expressing the TLR7^{P267L} variant, site-directed mutagenesis (SDM) was performed using TLR7 expression plasmid pcDNA3-TLR7-YFP (Addgene) and QuikChange II XL Site-Directed Mutagenesis Kit (Agilent) following manufacturer's instructions. SDM primers were designed using QuikChange Primer Design Tool (Agilent): TLR7 ^{P267L} Forward 5'-ACGGCGCACAAGGAAATAGGGCATTATAACAACGA-3' and Reverse 5'-TCGTTGTTATAATGCCCTATTTCCTTGTGCGCCGT-3'. The expression of YFP protein was removed by inserting the stop codon after TLR7 sequence by another SDM using primers: Forward 5'-TTCAAGGAAACGGTCTAAGAGATGGTGAGCAAG-3' and Reverse 5'-CTTGCTCACCATCTCTTAGACCGTTTCCTTGAA-3'. PCR products were then used to transform DH5α competent E. coli bacteria (C2987I strain, New England BioLabs) following a protocol provided by the supplier.

TLR7 protein expression in HEK293T cell line by Western Blotting

HEK293T cell line (ATCC) were cultured in DMEM culture media (1X, Gibco) supplemented with 10% FCS, 1% L-Glutamine (Sigma Aldrich) and 1% Penicillin-Streptomycin (Sigma Aldrich) at 37°C.

Cells were transiently transfected with 200ng of plasmid DNA pcDNA3-TLR7^{WT} or pcDNA3-TLR7^{P267L} and 300ng pcDNA3-empty using Fugene HD Transfection Reagent (Promega) according to manufacturer's instructions and incubated at 37°C for 48h.

Transfected HEK293T cells were 48h post-transfection washed in PBS and lysed in lysis buffer for 15min ([50 mM Tris-HCl (pH 7.5), 150 mM NaCl, 1% Nonidet P-40, 0.1% SDS, 0.5% Na-Deoxycholate] containing 100 mM dithiothreitol (Merck), 1x complete protease inhibitor cocktail (Roche), 1x PhosSTOP phosphatase inhibitors (Roche), and 1x NuPAGE Loading Buffer (Thermo Fisher Scientific)). Lysates were denatured at 75°C for 10 min before being subjected to 4-12% tris-glycine polyacrylamide gel (Thermo Fisher Scientific) electrophoresis in 1x SDS NuPAGE MOPS Running Buffer (Thermo Fisher Scientific). Proteins were transferred to 0.45-mm Immobilon-P polyvinyl difluoride membranes (Thermo Fisher Scientific) in 1x NuPAGE Tris-Glycine Transfer Buffer supplemented with 20% Methanol. Membranes were blocked for 60 min in 5% bovine serum albumin in tris-buffered saline with 0.1% Tween (TBS-T) buffer before overnight immunostaining at 4°C with rabbit anti-human TLR7 (Cell Signaling, 5632S, 1:1000) and rabbit anti-human GAPDH antibodies (Cell Signaling, 8884S, 1:5000). Membranes were washed in TBS-T and stained with secondary antibody anti-rabbit IgG HRP-linked (Cell Signaling, 7074S, 1:5000) for 1hour at RT. Membranes were developed with Immobilon ECL Ultra Western Substrate solution (Merck), imaged on a LI-COR Odyssey Fc (LI-COR) and Image Studio software was used for analysis.

Dual Luciferase NF-kB reporter assay

HEK293T cells were co-transfected using Fugene with 200ng of plasmid DNA pcDNA3-TLR7^{WT} or pcDNA3-TLR7^{P267L} and 180ng pcDNA3-empty, together with 100ng FireFly luciferase 4xNF-kB reporter plasmid (Addgene) and 20ng Renilla luciferase pRL-TK plasmid (Addgene). 48h post-transfection, cells were stimulated for additional 24h with 0.5-1ug/ml TLR7 ligands CL307 (InvivoGen, tlrl-c307) and CL097 (InvivoGen, tlrl-c97). Subsequently, cells were lysed for 20min at RT with 1X Passive Lysis Buffer (Promega), transferred to a Nunc F96 MicroWell White Polystyrene 96-well Plate (Thermo Scientific). Dual-Luciferase Reporter Assay System (Promega) was used following the standard protocol supplied by the manufacturer to measure the luminescence using Tecan23 SparkR M20 multimode microplate reader (Tecan). Detected FireFly luminescence signal was normalised to Renilla signal.

Statistical Analysis

GraphPad Prism software (version 9, GraphPad Software) was used for graph assembly and statistical analysis, error bars display mean with standard deviation (SD). 2way ANOVA test was applied to determine statistical significance. Data was determined statistically significant at $p < 0.05$ and defined as *($p<0.05$), **($p<0.01$), ***($p<0.001$), ****($p<0.0001$).

Data Availability

A single XLS file containing values for all data points in the graphs for all Figures in Manuscript and Supplement is available and is reported in the Supporting Data Values file.

Sex as a Biological Variable

This report describes a unique individual with TLR7 GOF who is female, while controls were drawn from both sexes equally. Our focus was not the influence of sex upon the penetrance or expressivity of TLR7 gain-of-function; however, the fact that this is an X-linked gene may well be pertinent to this issue. TLR7 is known to undergo incomplete Lyonization (7) which has been proposed to contribute to the greater risk of autoimmune disease in females (8). All 5 patients with TLR7 GOF in the original report of Brown et al. were female (3), but one of four was male in the recent case series by David et al. (9).

Supplementary References

7. Souyris M et al. TLR7 escapes X chromosome inactivation in immune cells. *Sci Immunol* 2018;3(19):eaap8855.

8. Souyris M, Mejía JE, Chaumeil J, Guéry J-C. Female predisposition to TLR7-driven autoimmunity: gene dosage and the escape from X chromosome inactivation. *Semin Immunopathol* 2019;41(2):153-164.

9. David C et al. Interface Gain-of-Function Mutations in TLR7 Cause Systemic and Neuro-inflammatory Disease. *J Clin Immunol* 2024;44(2):60.

British
Female Infant

13 months — **Anti-NMDAR encephalitis: developmental regression and movement disorder**

- refractory to steroids, plasmapheresis and rituximab
- responsive to high dose intravenous immunoglobulin (IVIg)
- delayed febrile reaction to rituximab after the 1st dose and a more severe reaction (Stevens-Johnson like) after the 2nd dose

23 months — **cSLE: intermittent fevers, rash, marked pallor and lethargy**

- large pericardial effusion, hepatosplenomegaly and ascites
- laboratory values reflective of SLE (see Supplementary Table 1)
- treated with high-dose corticosteroids, IVIg and mycophenolate mofetil, with partial response

25 months — **Neurovasculitis: status epilepticus, encephalopathy, livedo reticularis**

- MRI Brain: inflammatory vasculitis and focal haemorrhagic infarcts
- treated with corticosteroid pulse, B cell depletion using ofatumumab, IVIg, and a single dose of cyclophosphamide
- interferon stimulated gene signature as well as neutrophil transcriptional gene signature remained pathologically elevated

28 months — **WES – identified *de novo* heterozygous p.P267L in *TLR7* gene**

30 months

- JAK inhibitor baricitinib substituted for MMF
- Continuing low dose corticosteroid and intermittent Ofatumumab, enabling sustained remission

35 months — **Conditioning with anti-thymocyte globulin, ofatumumab, treosulfan, thiotepa and fludarabine**

36 months — **Maternal TCRαβ/CD19-depleted haploidentical peripheral blood HSCT**

dose CD34+ stem cells of 12.5 $x10^6$/kg

- Neutrophil and platelet engraftment on post-transplant days +13 and +17 respectively
- No major peri-transplant complications

45 months — **SARS-COV2 infection triggers symptomatic autoimmune haemolytic anaemia**

- Treated with blood transfusion and immunomodulation (corticosteroids, IVIg, sirolimus)

52 months — **Overall excellent progress**

- Remains DAT-positive, with evidence of grumbling haemolysis that occasionally flares, requiring additional immunomodulation

Supplementary Figure S1. Patient's progress timeline.

Supplementary Table 1. Patient's Laboratory Values

	**Presentation with cSLE (23 mo)**	**Presentation with neuro-vasculitis (25 mo)**	**Reference range**
Haemoglobin (g/L)	46	108	110 – 140
White cell count (x 10^9)	29	7	6 - 16
Neutrophils (x 10^9)	23	5	1 – 7
Lymphocytes (x 10^9)	4.6	1.1	3 – 11
Platelets (x 10^9)	133	290	150 – 400
Albumin (g/L)	16	26	35-50
Complement<br>C3 (g/L)<br>C4 (g/L)	0.25<br><0.05	0.85<br>0.1	0.9 – 1.8<br>0.1 – 0.4
CRP (mg/L)	34	17	<6.0
ESR (mm/Hr)	>120	25	<10
Anti-dsDNA (IU/mL)	>189.5	59	<27
Anti-nuclear antibody	Positive	Negative	
Antiphospholipid antibodies	β2GP1 positive	Negative	
ENA screen	Anti-Smith positive<br>Anti-RNP positive	Historic anti-Smith, anti-RNP	
DAT screen	Positive	-	
Ultrasound Abdomen	Hepatosplenomegaly	-	
Echocardiogram	Large pericardial effusion	-	
MRI Brain	-	-Extensive bilateral hyperintensity<br>-Focal infarcts	

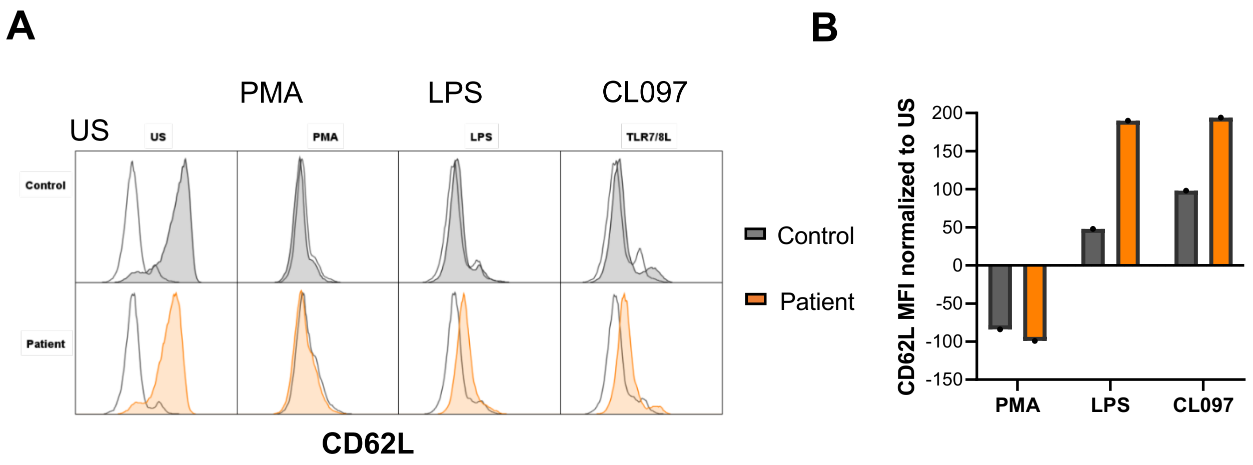

Supplementary Figure S2. Normal CD62L shedding in patient. A) Flow cytometry histograms of CD62L in unstimulated (US) and stimulated cells with PMA, LPS and TLR7/8 CL097 ligand. Empty line: isotype control. Shaded line: CD62L staining. B) Quantification of CD62L MFI normalized to unstimulated cells.

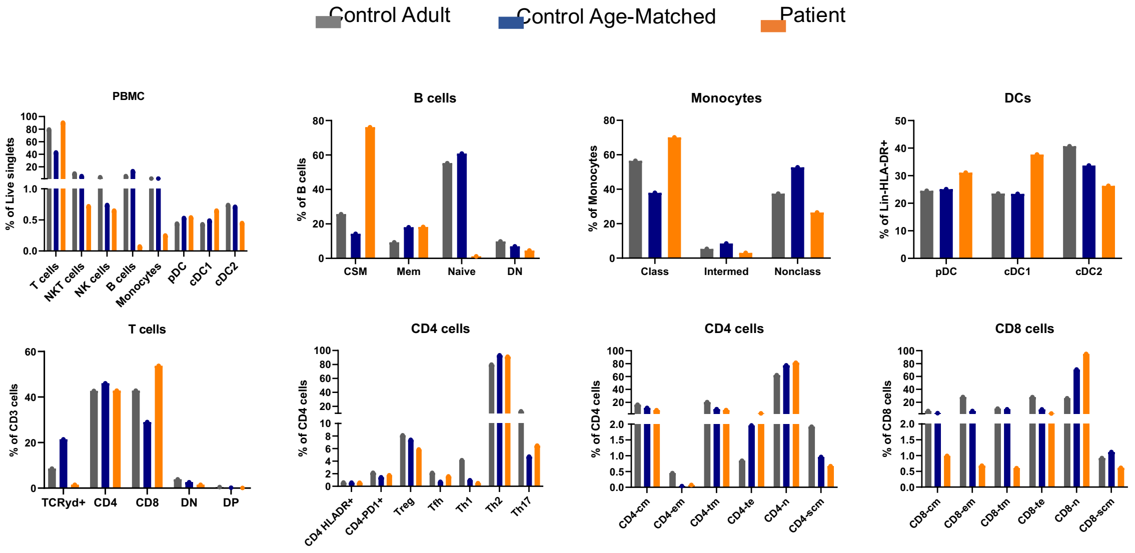

Supplementary Figure S3. Deep immunophenotyping of patient's PBMC.
