# Supplementary figures and images for: A de novo TLR7 gain-of-function mutation causing severe monogenic lupus in an infant
Source: J Clin Invest. 2024 May 16;134(13):e179193. doi: 10.1172/JCI179193 (PMC11213501; doi:10.1172/JCI179193)

Unedited gel images for Spegarova Stremenova et al (Fig 1 H)

GAPDH:

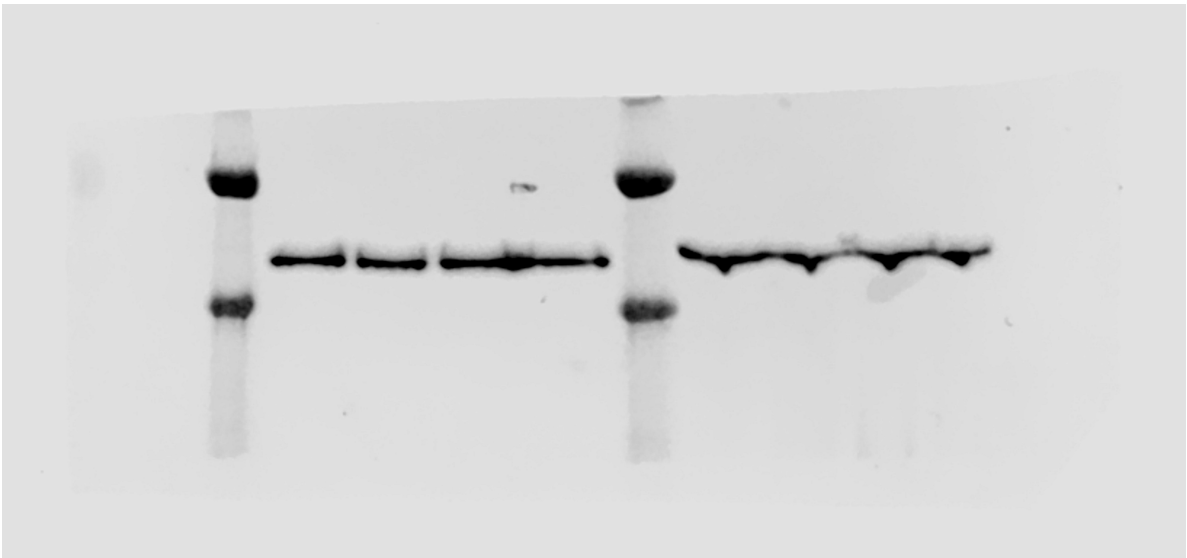

TLR7:

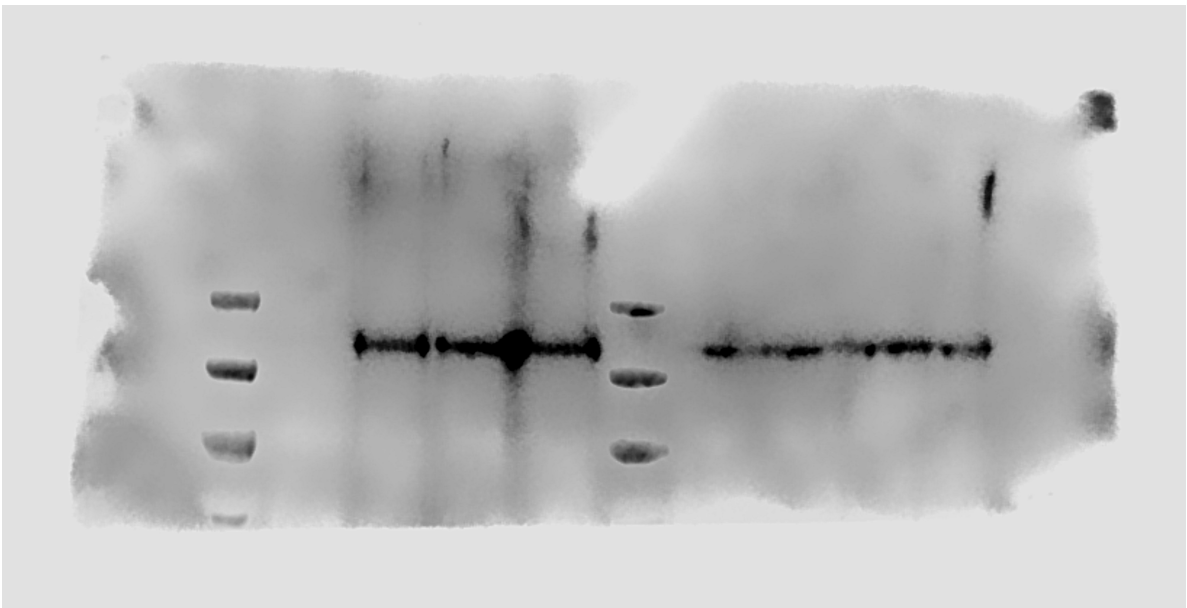

Supplement: Unedited blot and gel images [file jci-134-179193-s121.pdf]
